# *Senna occidentalis* (L.) Link root extract inhibits *Plasmodium* growth in vitro and in mice

**DOI:** 10.1186/s12906-023-03854-8

**Published:** 2023-03-06

**Authors:** Simeon Mogaka, Halkano Molu, Esther Kagasi, Kenneth Ogila, Rebeccah Waihenya, Faith Onditi, Hastings Ozwara

**Affiliations:** 1 Department of Tropical and Infectious Diseases, Institute of Primate Research, P.O Box 24481, Karen, Nairobi, 00502 Kenya; 2 Department of Zoology, Jomo Kenyatta University of Agriculture and Technology, P.O Box 62000-00200, Nairobi, Kenya; 3 Laboratory of Malaria Immunology and Vaccinology, National Institute of Allergy and Infectious Diseases, National Institutes of Health, Rockville, MD USA

**Keywords:** Ethnomedicine, In vitro, In vivo, Malaria, Plasmodium, *Senna occidentalis*

## Abstract

**Background:**

*Senna occidentalis* (L.) Link has been used worldwide in traditional treatment of many diseases and conditions including snakebite. In Kenya, a decoction from the plant roots taken orally, is used as a cure for malaria. Several studies have demonstrated that extracts from the plant possess antiplasmodial activity, in vitro. However, the safety and curative potency of the plant root against established malaria infection is yet to be scientifically validated, in vivo. On the other hand, there are reports on variation in bioactivity of extracts obtained from this plant species, depending on the plant part used and place of origin among other factors. In this study, we demonstrated the antiplasmodial activity of *Senna occidentalis* roots extract in vitro, and in mice.

**Methods:**

Methanol, ethyl acetate, chloroform, hexane and water extracts of *S. occidentalis* root were tested for in vitro antiplasmodial activity against *Plasmodium falciparum,* strain 3D7. Cytotoxicity of the most active solvent extracts was determined using 3-(4,5-dimethylthiazol-2-yl)-2,5-diphenyltetrazolium bromide (MTT) assay and the curative potency in *Plasmodium berghei* infected mice evaluated by Rane’s test.

**Results:**

All of the solvent extracts tested in this study inhibited the propagation of *P. falciparum,* strain 3D7, in vitro, with polar extracts being more active than non-polar ones. Methanolic extracts had the highest activity (IC_50_ = 1.76) while hexane extract displayed the lowest activity (IC_50_ = 18.47). At the tested concentrations, methanolic and aqueous extracts exhibited high selectivity index against *P. falciparum* strain 3D7 (SI > 10) in the cytotoxicity assay. Further, the extracts significantly suppressed the propagation of *P. berghei* parasites (*P* < 0.05) in vivo and increased the survival time of the infected mice (*P* < 0.0001).

**Conclusions:**

*Senna occidentalis* (L.) Link root extract inhibits the propagation of malaria parasites in vitro and in BALB/c mice.

**Supplementary Information:**

The online version contains supplementary material available at 10.1186/s12906-023-03854-8.

## Background

Malaria continues to be a public health challenge particularly in Sub-Saharan Africa [1, 2]. There were an estimated 247 million malaria cases and 619,000 deaths globally in 2021 [3]. Although malaria control has proven to be very effective when vector control is combined with prompt treatment, drug resistance to the insecticides used in vector control and antimalarial drugs pose a significant challenge to malaria control programs [1, 4–7]. Furthermore, some of the available antimalarial drugs have been linked to adverse side effects such as headache, diarrhea, nausea, pruritus, anxiety, seizures and hair discoloration. Long-term use of the drugs may lead to heart, eye and neuron defects [8].

Currently, the World Health Organization (WHO) recommends combining artemisinin and its derivatives with other antimalarial drugs in combination therapy [2, 9, 10]. This approach has proven to be effective in the treatment of *P. falciparum* malaria in various parts of the world. However, Plasmodium malaria resistance to current first line drugs, including artemisinin, has been reported [2, 10–14]. As such, priority should be given to the discovery and development of antimalarial drugs with novel and distinct mechanisms of action.

Medicinal plants are a rich source of untapped antimalarial activities [1, 2, 4, 15]. This is supported by the successful discovery of Artemisinin, Quinine and their derivatives as effective antimalarial drugs [16, 17]. *Senna occidentalis* (L.) Link is a widely used plant in traditional medicine throughout the world [18]. This plant is frequently used to treat a variety of infections and other conditions like snake and insect bites [18–21]. In traditional medicine, all parts of the plant are used [19]. The plant possesses antioxidant, nephroprotective and hepatoprotective activity, immunomodulatory activity, antidiabetic activity, analgesic and antipyretic activity, antianxiety, antidepressant and antimutagenic activity as well as antibacterial and antifungal activity [22–25].

In Kenya, *Senna occidentalis* root decoction is taken orally three times daily for 3 to 4 days by the Luhya, Digo and Duruma communities for treatment of malaria [26–28]. Previous in vitro studies have shown that *S. occidentalis* extract is active against *P. falciparum.* In one of the studies, *S. occidentalis* inhibited the growth of chloroquine sensitive (strain 3D7) and resistant (strain Dd2) *P. falciparum* with half-maximal inhibitory concentration (IC_50_) values of 65 μg/ml and 110 μg/ml respectively [29]. In a different study [30], an ethanolic extract of the plant similarly suppressed the growth of chloroquine sensitive *P. falciparum* (IC_50_ < 3 μg/ml), in vitro. In yet another study, *S. occidentalis* extract showed in vitro activity against chloroquine sensitive *P. falciparum*, strain 3D7, (IC_50_ = 48.80 μg/ml) and the resistant parasites, stain INDO, (IC_50_ = 54.28 μg/ml) [18]. In a more recent study [31], an extract from the plant exhibited in vitro activity against *P. falciparum* (3D7 strain) with an IC_50_ value of 3.37 μg/ml. The variation in IC_50_ values obtained in these studies could be due to differences in geographical origin of the plant, parts of the plant used, season of the year when plant material collection was done, methods of preparation and storage [25, 32, 33], or differences in assay protocols [34–36]. This current study sought to investigate further the antimalarial potency of the plant extract against *P. falciparum,* in vitro and *P. berghei*, in vivo.

## Methods

### Ethical clearance

Approval to conduct this study was granted by the Institute of Primate Research (IPR) Institutional Scientific and Ethics Review Committee (ISERC/02/18). The plant material used in this study was collected according to the WHO guidelines on Good Agriculture and Collection Practices (GACP) for medicinal plants [37]. The handling and care of experimental mice was done in compliance with the international guidelines on the care and use of laboratory animals. In addition, the study was done based on the Animal Research: Reporting of In Vivo Experiments (ARRIVE) guidelines. The Institute of Primate Research facility is registered by the National Commission for Science, Technology and Innovation (NACOSTI), Kenya and accredited by the Association for Assessment and Accreditation of Laboratory Animal Care (AAALAC) International.

### Plant collection, extraction and processing

*Senna occidentalis* roots were collected from Migori county (0.9366^o^ S, 34.4198^o^ E), Kenya in the months of August and September. This plant was identified by Mr. Jonathan Ayayo, a taxonomist of the National Museums of Kenya (NMK), and a voucher specimen (38/81) deposited at East African Herbarium of NMK for future reference. Further, the plant name was verified with http://www.theplantlist.org on 10/05/2022.

The collected plant roots were air dried in the shade, ground into powder and stored in airtight plastic containers at 4 °C until extraction.

Hexane, chloroform, ethyl acetate and methanol, in their absolute forms, as well as distilled water were used for extraction by maceration. For organic solvents, the plants root powder was macerated separately with the solvents for 48 hours in an orbital shaker and a filtrate obtained (Whatman No. 1 filter paper). For water, the roots powder was soaked in double distilled water for 24 hours. In addition, a decoction was prepared by boiling the roots powder in double distilled water for 30 minutes at a mean temperature of 95 °C [38]. Filtration of aqueous extracts was done through a cotton wool plug followed by filtration (Whatman No. 1 filter paper). The aqueous decoction was included to mimic the local’s traditional method of preparing the antimalarial therapy. The filtrates were concentrated by rotary vaporization (BÜCHI R-200 rotary evaporator) at 50 °C and reduced pressure for organic solvents, and lyophilization (NANBEI freeze dryer: NBJ-10-1, Zhengzhou, China) for aqueous solutions. Upon drying, the extracts were stored in sealed sample bottles at 4 °C until needed.

Plant secondary metabolites are excellent predictors of their bioactivity potential [39]. In order to predict the bioactivity of *S. occidentalis* roots extract, .standard procedures were used to screen the extracts for the presence of saponins, tannins, alkaloids, flavonoids and sterols [40].

### *Plasmodium falciparum* propagation and extracts preparation

*Senna occidentalis* root extracts were tested in vitro for antiplasmodial potency using *P. falciparum,* strain 3D7 obtained from Kenya Medical Research Institute (KEMRI) repository. A modified version of a previously used method [41] was utilised to establish a continuous culture of these malaria parasites. In summary, the parasites were propagated at 37 °C in blood group O+ red blood cells (RBCs) maintained in RPMI 1640 growth medium (Life technologies Ltd., Paisley, UK) supplemented with 1 M 4-(2-hydroxyethyl)-1-piperazine ethanesulfonic acid (HEPES), (Gibco, Life technologies Ltd., Paisley, UK), 1 M Sodium hydroxide (EMD Millipore corporation, Darmstadt, Germany), 20% D-glucose (PANREAC QUIMICA SA Barcelona, Spain), 200 mM L-glutamine (Gibco, Life technologies Ltd., Paisley, UK), 10% human serum (group O+) and a gas mixture of 90% N_2_, 5% O_2_ and 5% CO_2_. The culture was refreshed every 48 hours at 3% haematocrit.

Dilutions of the extract and standard drug were carried out in the same manner as described [42], with minor modifications. To make a stock solution (5 mg/ml), each extract was first dissolved in dimethyl sulfoxide (DMSO), vortexed and RPMI 1640 (incomplete medium) added to the required volume. For extract combinations, the extracts were weighed and blended in a 1:1 ratio [43]. A 0.5 mg/ml stock solution of pyrimethamine was also prepared.

### In vitro growth inhibition assay

Growth inhibition of *P. falciparum* malaria parasites by *S. occidentalis* root extract was assessed as described [44], with minor modification. A ten-fold dilution was prepared from the stock solutions using complete RPMI 1640 and serially diluted 7-fold across a 96-well cell culture plate. This provided a dose-titration range of 250 μg/ml to 1.95 μg/ml for the extracts and 25 μg/ml to 0.20 μg/ml for pyrimethamine. Sorbitol synchronized ring stage parasitized RBCs (1% parasitemia) suspended in complete RPMI at 3% hematocrit was then added to respective wells*.* Non-infected RBCs as well as DMSO were also included in the assay as controls. The plates were incubated at 37 °C. After 72 hours, thin smears were prepared and parasites quantified by light microscopy. Percentage parasitemia suppression was computed and 50% inhibitory concentration (IC_50_) values determined for each extract [45, 46].

### Evaluation of the extract for cytotoxicity

To assess the in vitro cytotoxicity of *S. occidentalis* root extract, Vero cell line (sourced from KEMRI) was grown to confluent monolayer in Minimum Essential Medium Eagle (MEM) containing sodium bicarbonate and l-glutamine. The growth medium was supplemented with 1% pen-strep (Sigma), 1% HEPES (Gibco) and 10% Fetal bovine serum (FBS). The assay was performed as previously described [47, 48]. Briefly, 10,000 Vero cells contained in 100 μl cell suspension were seeded onto each well of a 96-cell culture plate and incubated for 24 hours at 37 °C in a 5% CO_2_ incubator to allow the cells achieve a layer of > 90% confluence. The cells were exposed to 100 μl of the extracts for 48 hours, in concentration ranges of 250–3.91 and 25–0.39 μg/ml for the extracts and pyrimethamine, respectively. Pyrimethamine and DMSO (0.4%) exposed, as well as untreated cells were included as controls. Following 48 hours’ incubation period, 10 μl of MTT reagent (5 mg/ml) was added aseptically to each well, tapped gently to mix and incubated for 3 hours at 37 °C. All media was then aspirated from the wells and 100 μl DMSO added. Optical density (OD) readings as obtained by an ELISA reader were then used to compute the extracts’ 50% cytotoxicity concentration (CC_50_) and selectivity index (SI) as described elsewhere [48, 49].

### Experimental animals and rodent malaria parasites

In this study, male and female inbred BALB/c mice aged 8–9 weeks were used. The rodent facility at IPR provided the animals. They were housed in standard Macron type II cages within 12 hours’ dark/light cycle at 23 °C. Food and water was provided ad libitum according the IPR’s Animal Science Department standard operating procedures.

*Plasmodium berghei*, strain ANKA (sourced from KEMRI) was retrieved from the IPR repository and maintained in mice [50] for use in this study. This parasite strain was utilized because it causes severe disease in BALB/c mice with clinical characteristics similar to *P. falciparum* infection [51]. Despite the phylogenetic distance between rodent and human malaria parasites, *P. berghei* and the human malaria parasites possess conserved genes that have over time allowed their use in Peters’ model of antimalarial drug efficacy testing [52].

### Mice infection, treatment and monitoring of parasitemia and survivorship

A modification of the method by Ryley and Peters (1970) was used to evaluate the curative potency of the extract against *P. berghei* in mice [53, 54]. Mice were inoculated intraperitoneally with 1 × 10^6^*P. berghei* infected erythrocytes and assigned randomly to 5 experimental groups; A, B, C, D and E (5 mice per group). At approximately 5% parasitemia (day 5 post-infection), treatment was started. Groups A and B were treated with 200 mg/kg and 100 mg/kg of the methanolic extract, respectively. Group C was treated with 200 mg/kg of the aqueous extract. Group D was treated with 1 mg/kg pyrimethamine (Sigma – Aldrich Chemie, Steinheim, Germany). Group E (the placebo group) was administered with 1% DMSO in phosphate buffered saline (vehicle). The treatment was administered orally for 4 consecutive days. The oral route was chosen based on documented ethnomedical usage of the plant [28]. The mice were monitored for survival till day 30 post infection. Meanwhile, parasitemia suppression was determined. The amount of extract administered was informed by dose recommendations for in vivo administration of crude extracts [55] as well as previous antimalarial studies involving *Senna* family [5, 56].

### Determination of parasite growth suppression

The effect of the extract on *P. berghei* growth inhibition in mice was determined on days 6, 8, 10 and 12 post infection as described previously [53]. Tail blood was used to prepare thin smears that were fixed with absolute methanol and observed under × 100 lens of a light microscope. The number of parasitized red blood cells (pRBCs) was examined per at least 1000 RBCs. Percentage parasitemia and parasite suppression was then computed using the following formulas [57];

$$\%\; parasitemia = \frac{Number\; of\; infected\; RBC}{Total\; number\; of\; RBCs}\times 100$$

$$\%\;suppression=\frac{\%\;parasitemia\;in\;control\;group-\%\;parasitemia\;in\;treatment\;group}{\%\;parasitemia\;in\;untreated\;control\;group}\times\;100$$

### Determination of mean survival time

To assess the effect of the extract on survival time of the infected mice, the number of days that each mouse lived from the day of parasite inoculation to death was recorded in a 30 days’ period. The following formula was used to determine the mean survival time of each group [58];

$$MST=\frac{The\;sum\;of\;survival\;time\;(days)\;for\;the\;mice\;in\;a\;group}{The\;total\;number\;of\;mice\;in\;the\;group}$$

### Statistical analysis

The collected data was recorded as means ± standard error of the means (M ± SEM). In vivo parasite suppression was analyzed through Ordinary One-Way ANOVA followed by Tukey’s multiple comparisons test at 95% confidence level (alpha = 0.05). Log rank analysis was used to compare survival time of mice in the different treatment groups. Graph Pad prism (Version 7.00, California, USA) was used for the analyses.

## Results

### *Senna occidentalis* root extract possesses secondary plant metabolites

Plants with active phytomedicine properties possess secondary metabolites (Okokon et al., 2017). Methanol, ethyl acetate, chloroform, hexane and aqueous extracts of *S. occidentalis roots* were screened for presence of secondary metabolites. All extracts contained saponins, tannins, flavonoids, alkaloids and sterols. Aqueous decoction provided the highest percentage yield (7%) whereas hexane maceration provided the least yield (2%). The characteristics and yields of the extracts are as shown (Supplementary data 1). These extracts were subsequently assayed for biological activity against *Plasmodium* spp. especially suppression of malaria parasites growth.

### *Senna occidentalis* roots extract suppresses *plasmodium falciparum* growth, in vitro

Methanol, ethyl acetate, chloroform, hexane and aqueous roots extracts of *S. occidentalis* were tested for growth suppression potency against *P. falciparum*. The use of solvents with differing polarity would enable evaluation of antiplasmodial activity of both polar and nonpolar metabolites of the plant root. The results (Table 1) show that methanolic extract exhibited minimum IC_50_ value (IC_50_ = 1.76) whereas hexane extract exhibited maximum IC_50_ value (IC_50_ = 18.47). The parasite growth suppression levels are as shown (Supplementary data 2). The results imply that methanolic extract was 10-fold active against *P. falciparum* compared to hexane extracts. When the antiplasmodial activity of the other extracts was compared to that of hexane, the activity was 8, 4, 2.7 and 1.5-fold higher for aqueous macerate, ethyl acetate, aqueous decoction and chloroform respectively. These results suggest that polar extracts of *S. occidentalis* roots are more active against *P. falciparum,* in vitro*,* than non-polar ones. Furthermore, the findings demonstrate that both polar and non-polar metabolites of *S. occidentalis* roots are potent against *P. falciparum* in vitro.

**Table 1:** The 50% inhibitory concentrations, 50% cytotoxicity concentration and selectivity index values of the extracts

Type of extract/Control	IC_**50**_ (μg/ml) on 3D7	CC_**50**_ (μg/ml) on Vero cells	SI
Methanol	1.756	247	140
Aqueous macerate	2.283	1786	781
Methanol + Ethyl acetate	4.209	ND	
Ethyl acetate	4.728	ND	
Aqueous macerate+ ethyl acetate)	5.831	ND	
Aqueous decoction	6.831	ND	
Chloroform	12.130	ND	
Methanol+ Hexane	14.560	ND	
Aqueous macerate + hexane	17.650	ND	
Hexane	18.470	ND	
Pyrimethamine (positive control)	0.003		

IC_50_ (50% inhibitory concentrations); CC_50_ (50% cytotoxicity concentration); SI (selectivity index); ND (Not determined)

### *Senna occidentalis* roots extract is non-toxic to Vero cells, in vitro

Growth inhibitory effects observed in antiplasmodial assays may result from general toxicity of the substances being tested rather than specific activity on the *Plasmodium* parasites [49, 59]. The cytotoxicity of methanol and aqueous extracts were assessed using 3-(4,5-dimethylthiazol-2-yl)-2,5-diphenyltetrazolium bromide (MTT) assay. These two extracts were selected for cytotoxicity evaluation because they showed greater antiplasmodial activity in the in vitro assay and, were as such, candidates for pre-clinical analysis for antiplasmodial activity in animal models. Both the methanolic and aqueous extracts exhibited selectivity indices greater than 10 (Table 1), an indication of high selectivity for malaria parasites [48]. This observation implies that *S. occidentalis* root extract is non-toxic to animal cells at the tested concentrations.

### *Senna occidentalis* roots extract suppresses *plasmodium berghei* parasitemia in mice

**Fig. 1:**
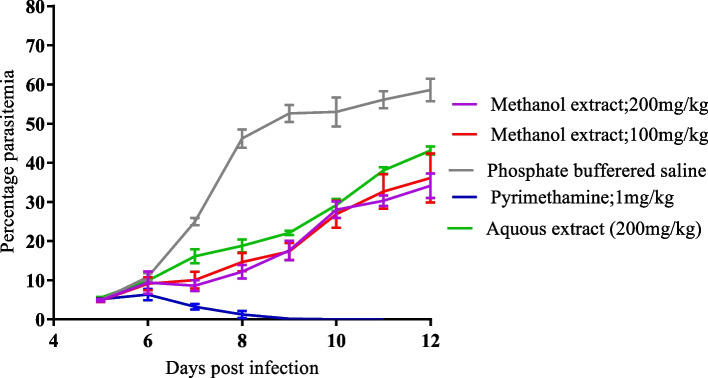
Parasitemic profile of pyrimethamine-sensitive *Plasmodium berghei* 7 days upon extract treatment, in comparison with controls

The *P. berghei*-BALB/c mouse model was utilized to assess the antiplasmodial activity of *S. occidentalis* polar extracts in situ in an animal system. The effect of *S. occidentalis* extract on *P. berghei* parasitemia suppression was determined by monitoring parasite multiplication in the infected mice upon treatment with the extract. At a dose of 200 mg/kg body weight, both the methanolic and aqueous extracts significantly suppressed *P. berghei* growth (*P* < 0.05) on days 6, 8, 10 and 12, relative to the phosphate buffered saline (placebo) group. At a dose of 200 mg/kg body weight, the inhibitory activity of methanol extract was not significantly different from that of aqueous extract (*P* > 0.05), except on day 6 post infection when the methanolic extract depicted higher activity (*P* = 0.03). The suppressive activity of 100 mg/kg methanol extract was not significantly different from that of 200 mg/kg aqueous extract (*P* > 0.05). Likewise, the suppressive activity of 100 mg/kg methanolic extract was not significantly different from that of 200 mg/kg extract of the same solvent (*P* > 0.05). This suggests using 100 mg/kg methanolic extract as optimum for use in mice. Parasite multiplication increased steadily in the placebo group until day 8 post-infection when it began to lag. This was indicative of reticulocyte depletion resulting in fewer cells available for malaria parasite infection [60, 61]. The pyrimethamine control group achieved 100% parasite clearance by day 9 post infection. Overall, these findings show that aqueous and methanolic extracts of *S. occidentalis* roots suppress *P. berghei* propagation, in vivo and that the aqueous extract is as effective as the methanolic extract at the tested concentrations. The parasite suppression levels for the different treatment groups are as shown in Table 2. Figure 1 shows parasitemic profiles of *P. berghei* in mice treated with the extract and the controls.

**Table 2:** Parasitemia and growth suppression (%) of *Plasmodium berghei* in mice upon treatment

Treatment	Parasitemia (Mean ± SEM) and suppression (%)			
	Day 6	Day 8	Day 10	Day 12
A	9.41 ± 2.82 (13.11*)	12.18 ± 1.72 (73.63*)	28.03 ± 2.13 (47.11*)	34.13 ± 5.14 (41.74*)
B	9.12 ± 1.68 (15.79*)	14.62 ± 2.36 (68.35*)	26.87 ± 3.46 (49.30*)	36.13 ± 6.29 (38.32*)
C	9.89 ± 0.65 (8.68*)	18.77 ± 1.64 (59.36*)	29.22 ± 1.52 (44.87*)	43.15 ± 1.04 (26.34*)
D	6.35 ± 1.45 (41.37*)	1.26 ± 0.17 (97.27*)	0.00 ± 0.00 (100*)	0.00 ± 0.00 (100*)
E	10.83 ± 0.93 (0*)	46.19 ± 2.34 (0*)	53.0 ± 3.73 (0*)	58.58 ± 2.9 (0*)

A (Methanol extract: 200 mg/kg body weight); B (Methanol extract: 100 mg/kg body weight); C (Aqueous extract:200 mg/kg body weight); D (Pyrimethamine:1 mg/kg body weight); E (Phosphate buffered saline); Suppression percentage (*)

### *Senna occidentalis* roots extract enhances the survival time of *plasmodium berghei* infected mice

**Fig. 2:**
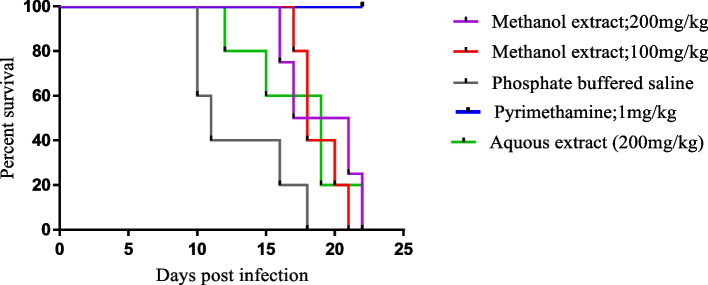
Survivorship curves of *Plasmodium berghei* infected upon treatment with the extracts

The effect of *S. occidentalis* extract on the survival of *P. berghei* infected mice was assessed by monitoring survival time of the extract-treated animals for 30 days, post- infection. The extract prolonged the survival time of the infected mice in the range of 4 to 6 days, which is approximately 0.3- to 0.5-fold extension of survival time relative to the placebo-treated animals. Treatment with the standard drug, pyrimethamine, resulted in 100% survival of the infected mice. When compared to the placebo group, the increase in survival time of the extract treated mice was significant (*P* < 0.0001). The mean number of days survived by the infected animals post infection is as shown in Table 3. Figure 2 represents survivorship curves for the animals in the different treatment groups.

**Table 3:** Survival time of *Plasmodium berghei* infected mice upon treatment with the extracts and controls

Treatment/control	Cumulative survival time (days)	Survival time in days (Mean ± SEM)
A	95	19 ± 1.14
B	94	18.8 ± 0.73
C	65	17.4 ± 1.75
D	150	30 ± 0.00
E	87	13 ± 1.67

A (Methanol extract: 200 mg/kg body weight); B (Methanol extract: 100 mg/kg body weight); C (Aqueous extract:200 mg/kg body weight); D (Pyrimethamine:1 mg/kg body weight); E (Phosphate buffered saline)

## Discussion

Natural products, especially medicinal plants, have been used to treat malaria or manage conditions associated with it in various parts of the world [5, 7]. We show, in this study, that *S. occidentalis* root extract inhibits malaria parasites growth in vitro and in mice*.* Our findings augment various studies that have illustrated the antimalarial potency of medicinal plants, as reviewed [62–64]. The documented ethnomedical use of the plant root as a cure for malaria in Kenya influenced the selection of the plant for antiplasmodial in vitro evaluation [27, 28]. This ethnopharmacological approach to drug development has been associated with high success rates of discovering active compounds from natural sources [49]. It is also a cost effective and time saving approach compared to mass screening of plants for specific biological activity [65, 66].

To obtain polar and nonpolar root extracts of *S. occidentalis* for evaluation of antimalarial potential, the plants roots powder was extracted with methanol, ethyl acetate, chloroform, hexane and water. Preliminary phytochemical analysis of the extracts revealed the presence of biologically important secondary metabolites, some of which have been linked to antimalarial activities of plant extracts [25, 39, 54, 67, 68]. These findings paved way for further analysis of *S. occidentalis* extracts as antimalarials. A bioactivity guided extract fractionation will reveal the actual molecules containing antiplasmodial activity.

In this current study, the extracts were found to be active, in vitro*,* against *P. falciparum*. This malaria parasite species is the foremost contributor to the global burden of malaria [1, 4, 9]. Our findings revealed that antiplasmodial activity of *S. occidentalis* roots is polarity dependent with extracts from polar solvents showing higher activity than non-polar ones. This suggests that during extraction, the bioactive principles of the plant roots get localized more in polar solvents than non-polar ones. Extraction efficiency has been shown to be dependent on the method and solvent of extraction [69]. Differences in extraction temperature may account for the variation in bioactivity between aqueous macerate and decoction extracts. High temperatures lead to loss of thermolabile compounds or even transformation of the phytochemicals [69]. Overall, these findings imply that the antimalarial ingredients of the roots extract of *S. occidentalis* are mainly polar.

In this current study, *S. occidentalis* root extract showed no toxicity to Vero cells and was highly selective of *P. falciparum*. This is suggestive of possible safety of the extracts when used in in vivo models of antimalarial efficacy testing.

Drug interaction with malaria parasites is heavily influenced by the host biological system [34, 70]. In this current study, *S. occidentalis* root extract was evaluated for antiplasmodial activity in situ using the *P. berghei* mouse model of antimalarial drug efficacy testing. In this in vivo assay, use of human malaria parasites (*P. falciparum*), just like in the in vitro test, would have been more appropriate. However, we did not have access to an animal model for *P. falciparum* antimalarial drug efficacy testing. Alternatively, our laboratory has established protocols for in vivo antimalarial drug efficacy testing *using P. berghei* in a mouse model. The structure, life cycle and physiology of *P. berghei* compare with those of the human malaria parasites [71]. Accordingly, mouse specific parasite species have been used in Peters’ standard test to provide important data in support of antimalarial drug development [72, 73]. As such the *P. berghei* mouse model would provide valuable information regarding the in vivo antiplasmodial potential of the extract under investigation in the current study. The effect of *S. occidentalis* extract on *P. berghei* parasitemia was determined by monitoring parasite propagation in infected BALB/c mice upon treatment with the extract. The extract significantly suppressed *P. berghei* parasitemia in mice and increased the survival time of the infected animals. The longer survival time of mice treated with the extract corresponded with reduced parasitemia, suggesting that the extract may play a role in reducing the pathologic effects of the infection. A positive correlation between parasite density, disease severity and mortality due to malaria has been described [74, 75]. In the current study, we did not evaluate the extract for protective activity against malaria infection. The next step would, therefore, be the analysis of the antimalarial potency of *S. occidentalis* extracts in a prophylactic, as well as non-human primate model for a more complete preclinical testing.

The presence of secondary metabolites in the extract investigated herein may explain the growth suppressive activities of the extract against malaria parasites observed in this study. Plant secondary metabolites have been shown to induce malaria parasite death through diverse mechanisms. For instance, flavonoids have been shown to possess antioxidant potential and kill *Plasmodium* parasites by chelating nucleic acids of malaria parasites [38, 76]. It is also reported elsewhere that antioxidants can inhibit heme polymerization, and that unpolymerized heme is very toxic to *Plasmodium* parasites, hence kills malaria parasites [77]. More studies are required to determine the mechanism used by *S. occidentalis* root extract in suppressing *P. falciparum* and *P. berghei* parasites growth.

## Conclusions

Overall, our findings demonstrate that *Senna occidentalis* (L.) Link root extract has remarkable antimalarial activity, in vitro and in mice. In addition, the results show that polar extracts of *S. occidentalis* are more potent than non-polar ones, implying that polar extracts can be developed further into antimalarials. We recommend the isolation and identification of the bioactive entities and the establishment of the antimalarial mechanism of action of the extract.

## Supplementary Information

**Additional file 1.** Characteristics of *Senna occidentalis* roots extract and the percentage yields.

**Additional file 2.** Growth inhibitory activity of *Senna occidentalis* root extracts against *Plasmodium falciparum,* in vitro*.*

## Data Availability

The datasets supporting the conclusions of this article are included within the article. The raw data used for analysis can be availed by the corresponding author on request.

## Abbreviations

AAALAC: Association for Assessment and Accreditation of Laboratory Animal Care
ARRIVE: Animal Research: Reporting of In Vivo Experiments
ANOVA: Analysis of variance
DMSO: Dimethyl sulfoxide
FBS: Fetal bovine serum
IPR: Institute of Primate Research
ISERC: Institutional Scientific and Ethics Review Committee
KEMRI: Kenya Medical Research Institute
MEM: Minimum Essential Medium Eagle
NACOSTI: National Commission for Science, Technology and Innovation
RBCs: Red blood cells
RPMI: Roswell Park Memorial Institute
WHO: World Health Organization
CC_50_: 50% cytotoxicity concentration
IC_50_: 50% inhibitory concentration
