# Supplementary material for: Senna occidentalis (L.) Link root extract inhibits Plasmodium growth in vitro and in mice
Source: BMC Complement Med Ther. 2023 Mar 6;23:71. doi: 10.1186/s12906-023-03854-8 (PMC9987147; doi:10.1186/s12906-023-03854-8)
Supplement: Supplementary file 1 — Additional file 1. Characteristics of Senna occidentalis roots extract and the percentage yields. [file 12906_2023_3854_MOESM1_ESM.pdf]

Characteristics of *Senna occidentalis* roots extract and the percentage yields

Extraction method	Weight of plant material(g)	Colour of extract	Texture of extract	Weight of extract(g)	Percentage yield
Aqueous (decoction)	250	Brown	Powder	17.7	7.08
Aqueous (maceration)	250	Brown	Powder	15.98	6.39
Methanol (maceration)	250	Dark brown	Paste	14.24	5.7
Ethyl acetate (maceration)	250	Dark brown	Paste	8.73	3.49
Chloroform (maceration)	250	Dark brown	Paste	6.85	2.74
Hexane (maceration)	250	Dark brown	Paste	5.33	2.13
