# Supplementary material for: Senna occidentalis (L.) Link root extract inhibits Plasmodium growth in vitro and in mice
Source: BMC Complement Med Ther. 2023 Mar 6;23:71. doi: 10.1186/s12906-023-03854-8 (PMC9987147; doi:10.1186/s12906-023-03854-8)
Supplement: Supplementary file 2 — Additional file 2. Growth inhibitory activity of Senna occidentalis root extracts against Plasmodium falciparum, in vitro. [file 12906_2023_3854_MOESM2_ESM.pdf]

Growth inhibitory activity of *Senna occidentalis* root extracts against *Plasmodium falciparum, in vitro*

Type of extract	Percentage parasitemia (Mean±SEM)	Growth suppression (%) range	IC_{50} (µg/ml)
Methanol	0.46±0.03 to 1.23±0.02	82.44 to 53.05	1.756
Aqueous macerate	0.69±0.02 to 1.38±0.11	73.66 to 47.33	2.283
Methanol + Ethyl acetate	0.73±0.00 to 1.38±0.04	72.14 to 47.33	4.209
Ethyl acetate	0.75±0.07 to 1.39±0.06	71.37 to 46.95	4.728
Aqueous macerate+ ethyl acetate)	0.70±0.09 to 1.42±0.06	73.28 to 45.80	5.831
Aqueous decoction	0.74±0.04 to 1.53±0.01	71.75 to 41.60	6.831
Chloroform	0.74±0.06 to 1.49±0.00	71.76 to 43.13	12.130
Methanol+ Hexane	0.76±0.02 to 1.81±0.11	70.99 to 30.91	14.560
Aqueous macerate + hexane	0.78±0.00 to 2.05±0.00	70.22 to 21.75	17.650
Hexane	0.89±0.05 to 1.73±0.04	66.03 to 33.97	18.470
**Controls**			
Pyrimethamine	0.18±0.00 to 0.82±0.06	93.03 to 68.70	0.003
Infected RBCs only	2.62±0.02	-	-
Infected RBCs and DMSO	2.53±0.01	-	-

RBCs: Red blood cells; DMSO: Dimethyl sulfoxide
